# Comparison of Clopidogrel and Ticlopidine/*Ginkgo Biloba* in Patients With Clopidogrel Resistance and Carotid Stenting

**DOI:** 10.3389/fneur.2019.00044

**Published:** 2019-01-30

**Authors:** Jong-Won Chung, Suk Jae Kim, Jaechun Hwang, Mi Ji Lee, Jun Lee, Kyung-Yul Lee, Man-Seok Park, Sang Min Sung, Keon Ha Kim, Pyoung Jeon, Oh Young Bang

**Affiliations:** ^1^Department of Neurology, Samsung Medical Center Sungkyunkwan University School of Medicine, Seoul, South Korea; ^2^Department of Neurology, Kyungpook National University School of Medicine Kyungpook National University Chilgok Hospital, Daegu, South Korea; ^3^Department of Neurology Yeungnam University Medical Center, Daegu, South Korea; ^4^Department of Neurology, Gangnam Severance Hospital Yonsei University College of Medicine, Seoul, South Korea; ^5^Department of Neurology Chonnam National University Medical School, Gwangju, South Korea; ^6^Department of Neurology Busan National University Hospital, Busan, South Korea; ^7^Department of Radiology, Samsung Medical Center Sungkyunkwan University School of Medicine, Seoul, South Korea

**Keywords:** clopidogrel resistance, carotid stenosis, stroke, ischemia, ticlopidine, surrogate endpoint

## Abstract

**Background and Purpose:** Patients undergoing carotid artery stenting (CAS) who show low responsiveness to clopidogrel may have a higher risk of peri-procedural embolic events. This study aimed to compare the effectiveness and safety of clopidogrel and ticlopidine plus *Ginkgo biloba* in clopidogrel-resistant patients undergoing CAS.

**Methods:** In this multi-center, randomized, controlled trial, we used platelet reactivity test to select patients undergoing CAS who showed clopidogrel resistance, and compared treatments using clopidogrel and ticlopidine plus ginkgo. The primary outcome was the incidence of new ischemic lesion in the ipsilateral hemisphere of CAS. Detection of microembolic signal on transcranial Doppler was the secondary outcome. The clinical outcomes were also monitored.

**Results:** This trial was discontinued after 42 patients were randomized after preplanned interim sample size re-estimation indicated an impractical sample size. The primary endpoint occurred in 12/22 patients (54.5%) in the clopidogrel group and 13/20 patients (65.0%) in the ticlopidine–ginkgo group (*P* = 0.610). No significant differences in the presence of microembolic signal (15.0 vs. 11.8%, *P* = 0.580), clinical outcomes (ischemic stroke or transient ischemic attack, 0.0 vs. 5.5%; acute myocardial infarction 0.0 vs. 0.0%; all-cause death, 4.5 vs. 0.0%), or incidence of adverse events were found in the two groups. In terms of resistance to clopidogrel, treatment with ticlopidine–ginkgo significantly increased the P2Y12 Reaction Units (difference, 0.0 [−0.3–3.0] vs. 21.0 [6.0–35.0], *P* < 0.001).

**Conclusions:** In patients who showed clopidogrel resistance, ticlopidine–ginkgo treatment was safe and increased P2Y12 Reaction Units; however, compared to clopidogrel, it failed to improve surrogate and clinical endpoints in patients undergoing CAS. This multimodal biomarker-based clinical trial is feasible in neurointerventional research.

**Clinical Trial Registration:** http://www.clinicaltrials.gov. Unique identifier: NCT02133989.

## Introduction

Antiplatelet agents are used to prevent stent thrombosis and peri-procedural complications. The extent of inhibition of platelet function by aspirin and clopidogrel differs among individuals, and is related to recurrent cerebrovascular or cardiovascular events during the use of antiplatelet agents. While the Clopidogrel in High-risk patients with Acute Non-disabling Cerebrovascular Events (CHANCE) trial showed that in comparison with aspirin alone, combined treatment with clopidogrel and aspirin decreases the 90-day risk of stroke without increasing hemorrhage (1), it also showed that this effect was not observed in *CYP2C19* loss-of-function alleles (2).

Several studies have dealt with the association between genetic (e.g., *CYP2C19* loss-of-function alleles) and laboratory (e.g., point-of-care tests for platelet aggregation) features of clopidogrel resistance with cardiovascular events and stroke in subjects receiving clopidogrel (3–8). However, few clinical trials have been conducted to test antiplatelet strategies to overcome clopidogrel resistance in patients with stroke or in those who undergo carotid intervention. The use of P2Y12 receptor inhibitors other than clopidogrel, such as ticlopidine or novel thienopyridines, could be a possible strategy against clopidogrel resistance. The use of thienopyridine prodrugs with more rapid and consistent actions (ticlopidine and prasugrel have more pathways resulting in active metabolites and are not deactivated by de-esterification) or direct-acting P2Y12 inhibitors (cangrelor and ticagrelor) could be alternatives to the standard clopidogrel therapy (9). Although a recent clinical trial tested the role of ticagrelor over aspirin in patients with ischemic stroke/transient ischemic attack (TIA), further studies are required to determine the optimal candidate for this novel thienopyridine (10–12). Current guidelines do not recommend the use of novel thienopyridines in patients with stroke.

**Figure 1:**
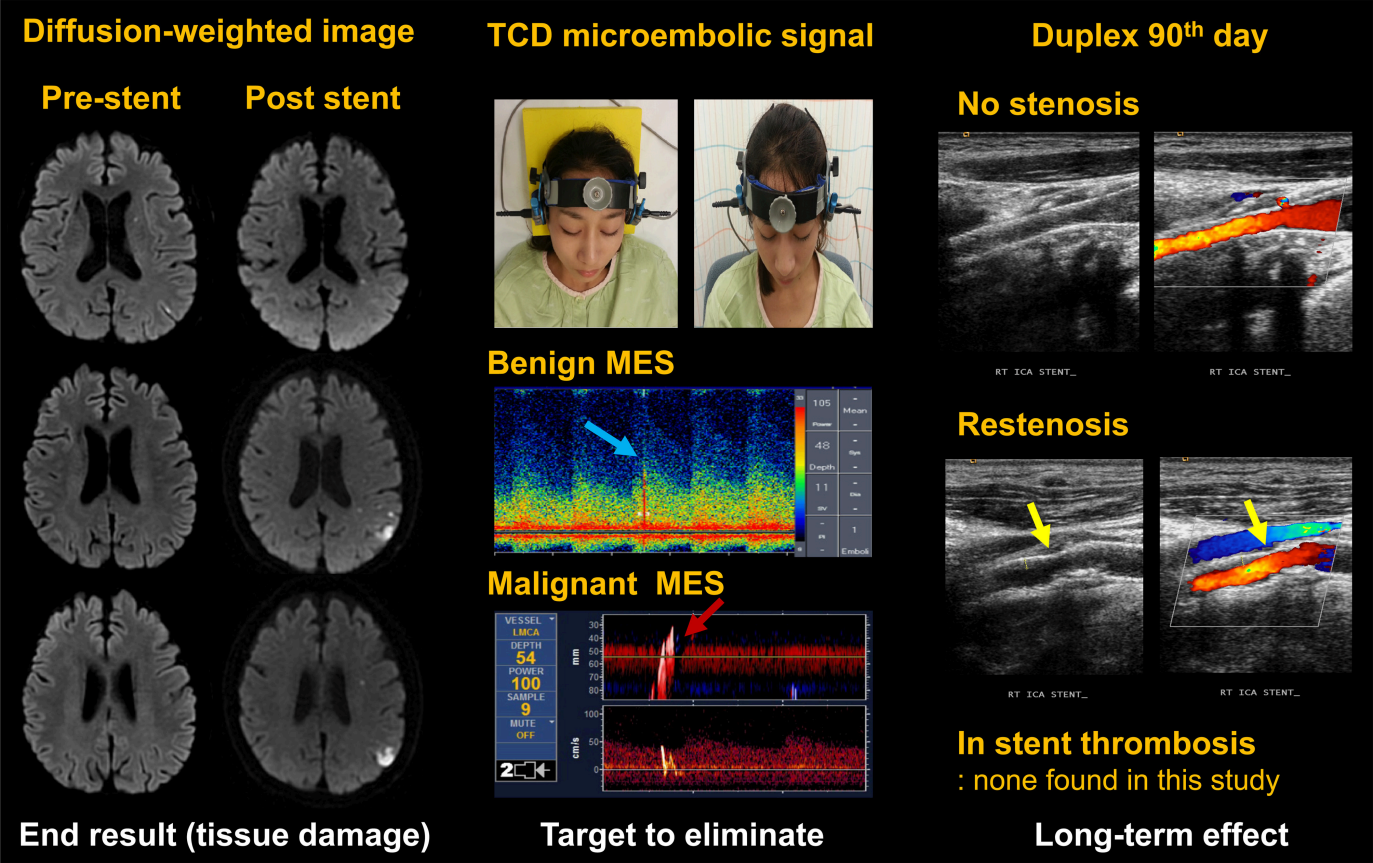
Measurement of laboratory outcomes. TCD, transcranial doppler; MES, microembolic signal. Written informed consent was obtained from the individual appearing in this figure (top, middle).

Biomarker-based diagnostic tests are increasingly being used as surrogate markers in clinical trials in cancer (13), cardiovascular disease (14), renal disease (15, 16), and neurological disorder (17, 18), and they might add important information from the neurointerventional point of view. In these clinical trials, instead of clinical events, laboratory outcomes were measured. In the present study, we selected three laboratory outcomes (Figure 1). First, ischemic brain lesions on diffusion-weighted images (DWIs), a marker of an increased risk of cerebrovascular events in the International Carotid Stenting Study (the recipient site) (19). Second, microembolic signals (MES) on transcranial duplex (TCD) ultrasound monitoring, which were related to clinical events and were used as markers for antiplatelet effects in patients with carotid and intracranial stenosis (migrating emboli) (20, 21). Third, the occurrence of restenosis on follow-up carotid duplex (the donor site). As an exploratory research, we tested the feasibility and usefulness of this multidisciplinary and comprehensive laboratory approach in a drug trial in the setting of a small number of patients with few clinical events.

The usefulness of ticlopidine in patients with loss-of-function CYP2C19 polymorphism carriers have been reported (22). In addition, an experiment demonstrated augmented antithrombotic and antiplatelet effects with ticlopidine and *Ginkgo biloba* (23, 24). This trial aimed to evaluate the efficacy and safety of ticlopidine plus *Ginkgo biloba* compared to clopidogrel using surrogate biomarkers in patients showing clopidogrel resistance who undergo carotid artery stent (CAS) placement.

## Patients and Methods

### Study Design and Oversight

The Clopidogrel Resistance and Embolism in Carotid Artery Stenting (CRECAS) trial is a multicenter prospective, randomized, open-label, blinded-endpoint trial. This study is registered with ClinicalTrials.gov (identifier, NCT02133989). Patients were enrolled from January 2014 through August 2017 at six sites in South Korea. The trial was approved by the appropriate national regulatory authorities and relevant ethnics committees at each participating site. All participants provided written informed consents.

The executive committee was responsible for the overall design, interpretation, and supervision of the trial, including the development of the protocol and any amendments. The executive committee was also responsible for ensuring the integrity of the data, analysis, and presentation of results. An independent data and safety monitoring committee reported to the executive committee, and regularly assessed the safety outcomes, overall study integrity, and study conduct. The sponsor had no influence or involvement in the design, conduct, analysis, and decision to terminate this trial. The sponsor was not part of the executive committee.

### Study Population

CRECAS-randomized patients had provided informed consent, were scheduled for stent implantation owing to carotid stenosis of 70% or more, and showed resistance to clopidogrel, defined by platelet inhibition rate < 20% measured by the VerifyNow system (Accumetrics, San Diego, CA, USA) in patients taking a clopidogrel dose of 75 mg/d for ≥7 days or 24 h after a loading dose of 300 mg for clopidogrel-naïve patients. Premedication with clopidogrel 75 mg for ≥7 days or 300 mg for ≥24 h showed similar platelet inhibition (25). All patients underwent brain MRI including DWI within 1 months prior to carotid stenting.

Patients were not eligible for participation in the trial if they were scheduled for other specific antiplatelet therapy or anticoagulation therapy or for carotid, cerebrovascular, or coronary revascularization that would require discontinuation of the study treatment within 60 days after randomization. Similarly, patients unable to undergo MR imaging; those with hematologic abnormalities including neutrophil count <1,500/μL, platelet count <100,000/μL, or AST/ALT >120 U/L; and those who failed to understand or comply with the study procedures or follow-up were excluded from the study.

### Treatment

Eligible patients were randomly assigned in a 1:1 ratio to one of two treatment groups, using random permuted blocks. Patients were 1:1 randomized to clopidogrel 75 mg daily or ticlopidine 250 mg/*Ginkgo biloba* 80 mg twice daily, together with a daily dose of aspirin 100 mg for a 60-day treatment period. At the end of 24 weeks of study treatment, patients were treated at the discretion of investigators and followed up.

Carotid stenting was performed according to the standardized method by skilled and experienced operators. CAS was performed as soon as possible in the clopidogrel group, and after 96 h of study medication in the ticlopidine–ginkgo group. All patients received high-intensity statins according to the current international guidelines before carotid stenting.

### Endpoints

The primary endpoint for the trial was the presence of new ischemic lesions in the ipsilateral hemisphere on DWI performed within 24 h after carotid stenting. The predefined secondary endpoints were the number and volume of new ischemic lesions on DWI performed within 24 h after carotid stenting, and total and malignant MES on TCD monitoring performed within 24 h after carotid stenting. TCD (Pioneer TC 8080; Nicolet Vascular, Madison, WI, USA) was used to monitor both middle cerebral arteries (MCAs) with insonation depths of 40–60 mm for microemboli using two 2-MHz probes fixed with a head frame (Marc 500; Spencer Technologies, Northborough, MA, USA). All MES' were automatically saved to the computer hard disk for review, and all analyses were performed blinded to individual patient details. Bilateral recordings were performed for 30 min, with the patients in a supine position. Patients who had ≥1 MES during the 30 min of TCD recording were classified as MES positive. In the present study, we also measured malignant MES, a larger-sized embolus requiring more clinical attention (26, 27). MES' with a relative energy index >1.0 were considered malignant.

Other exploratory endpoints included ischemic stroke/TIA, myocardial infarction, or death within 24 weeks after carotid stenting. The safety endpoints included puncture-site hematoma, hematological abnormalities, namely neutrophil count <1,500/μL, platelet count <100,000/μL, or AST/ALT level >120 U/L. We also measured the changes of clopidogrel resistance, by using the VerifyNow system. Clinical and laboratory follow-up was conducted on Day 1, Day 7, Week 4, and Week 24 after carotid stenting.

### Statistical Analysis

A total of 82 patients were required to detect a hazard ratio of 0.55, with a final two-sided significance level of 5 and 80% power, as observed in a previous observational study (28). To apply the 5% dropout rate, 86 patients were scheduled to be enrolled. A single pre-specified interim analysis for efficacy and futility was performed when half of the patients were enrolled for sample size recalculation or early discontinuation of the trial.

Statistical analyses were performed using PASW Statistics 18 (IBM, Chicago, IL, USA). Continuous variables are presented as means with standard deviations, and categorical variables are presented as frequencies and percentages. Variables were compared by independent *t*-test, chi-square test, Mann–Whitney *U*-test, and Fisher's exact test. A two-tailed value of *p* < 0.05 was considered statistically significant.

## Results

This trial was discontinued after prespecified interim analysis for futility and power re-estimation after enrolment of 42 participants. The primary endpoint, new ischemic lesions in the ipsilateral hemisphere of carotid stenting, was detected in 54.5% (12 of 22) of patients in the clopidogrel group and 55.0% (11 of 20) of patients in the ticlopidine–ginkgo group. Based on this result, the sample size was recalculated, and the estimated number of patients required to show significant difference in the two groups was 155,000 (*Z*-test with pooled variance statistics; power, 0.80; two-sided significance level, 0.05, with two independent proportions power analyses). PASS 12 (NCSS, Kaysville, Utah, USA) software was used to perform the sample size estimates. In addition, owing to the recently decreasing role of carotid intervention in patients with asymptomatic carotid stenosis, slow patient recruitment for the study was expected. The executive committee concluded that it was impractical to continue the study and decided to terminate it prematurely.

From June 2014 through October 2017, we recruited 42 patients, 22 of whom were assigned to the clopidogrel group and 20, to the ticlopidine–ginkgo group (Supplementary Table 1). The baseline characteristics of the IIT-analysis population are described in Table 1. Baseline characteristics were well-balanced between the groups, except that compared to the clopidogrel group (63.3%), more symptomatic (acute stroke or transient ischemic attack) patients were enrolled in the ticlopidine–ginkgo group (80.0%) (*P* = 0.037).

**Table 1:** Baseline characteristics.

	**Clopidogrel group (*n* = 22)**	**Ticlopidine–Ginkgo group (*n* = 20)**	***P*-value**
Age	70.2 ± 8.2	74.7 ± 8.8	0.096
**MALE**			
Body mass index	24.6 ± 3.3	23.7 ± 3.5	0.378
Systolic blood pressure	139.1 ± 20.1	135.0 ± 15.2	0.449
Diastolic blood pressure	79.1 ± 11.9	74.4 ± 9.4	0.159
Presenting symptom			0.037
Asymptomatic	8 (36.4)	4 (20.0)	
TIA	0 (0.0)	5 (25.0)	
Stroke	14 (63.6)	11 (55.0)	
Pre-treatment acute ischemic lesion	12 (54.5)	13 (65.0)	0.355
**MEDICAL HISTORY**			
Hypertension	17 (77.3)	16 (80.0)	0.565
Diabetes	14 (63.6)	8 (40.0)	0.111
Hyperlipidemia	10 (45.5)	8 (40.0)	0.483
History of CAD	2 (9.1)	3 (15.0)	0.453
History of stroke	9 (40.9)	6 (30.0)	0.34
**LABORATORY VALUES**			
Neutrophil	56.4 ± 15.0	58.3 ± 15.8	0.696
Platelet	221.1 ± 59.7	214.8 ± 31.2	0.668
AST	22.8 ± 7.2	19.5 ± 5.1	0.086
ALT	25.8 ± 19.5	15.5 ± 6.5	0.027
PRU, initial	239.4 ± 56.4	248.5 ± 47.4	0.576
PRU (%), initial	5.7 ± 6.4	6.5 ± 8.0	0.734
Carotid stenosis side			0.199
Right	17 (85.0)	13 (68.4)	
Left	3 (15.0)	6 (31.6)	
Carotid stenosis, degree	74.0 (70.0–88.75)	87.0 (80.0–90.0)	0.091
Carotid stenosis, ulcer	0 (0.0)	2 (10.5)	0.231
Carotid stenosis, contralateral	8 (40.0)	6 (31.6)	0.416

*TIA, transient ischemic attack; CAD, coronary artery disease; AST, aspartate aminotransferase; ALT, alanine aminotransferase; PRU, P2Y12 reaction units*.

**Figure 2:**
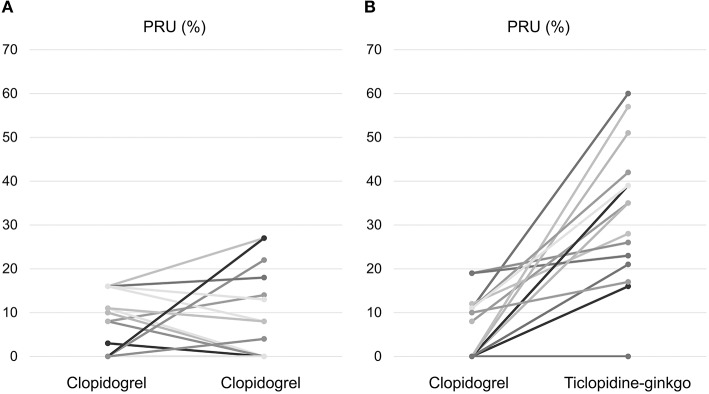
Changes in P2Y12 Reaction Units. Change in P2Y12 Reaction Units in the **(A)** clopidogrel and **(B)** ticlopidine–ginkgo group.

Before carotid stenting, acute ischemic lesions were found in 12 (54.5%) patients in the clopidogrel group and 13 (65.0%) patients in the ticlopidine–ginkgo group. The primary endpoint of new ischemic lesion(s) in ipsilateral carotid stenting occurred in 12 patients (54.5%) in the clopidogrel group and 11 patients (55.0%) in the ticlopidine–ginkgo group (*P* = 0.610). There were no significant differences between the groups with regard to microembolic signal and carotid duplex imaging. However, compared to persistent clopidogrel treatment, treatment with ticlopidine–ginkgo for a median duration of 7 days significantly improved P2Y12 Reaction Units (Figure 2). The details of laboratory outcomes are demonstrated in Table 2. During a median follow-up period of 178 days after carotid stenting, one patient in the ticlopidine–ginkgo group experienced stroke recurrence and one patient in the clopidogrel group died owing to lung cancer. None of the patients enrolled in the study experienced any hematologic or procedure-related adverse event. The clinical and adverse events are summarized in Table 3.

**Table 2:** Laboratory outcomes.

	**Clopidogrel group**	**Ticlopidine–Ginkgo group**	***P*-value**
Diffusion-weighted image	(*n* = 22)	(*n* = 20)	
Pre-treatment acute ischemic lesion	12 (54.5)	13 (65.0)	0.355
**POST-TREATMENT NEW ISCHEMIC LESION**			
Stenting side	12 (54.5)	11 (55.0)	0.610
No. of small lesions	1.0 (0.0–4.0)	1.0 (0.0–3.0)	0.447
Presence of large (>20 mm) lesion	2 (9.1)	2 (10.0)	0.659
Contralateral side	6 (27.3)	5 (25.0)	0.574
No. of small lesion	0.0 (0.0–1.0)	0.0 (0.0–0.75)	0.422
Presence of large lesion	0 (0.0)	0 (0.0)	N/A
Posterior circulation	2 (9.1)	1 (5.0)	0.537
Microembolic signal	(*n* = 20)	(*n* = 17)	
Presence	3 (15.0)	2 (11.8)	0.580
Malignant MES	0	0	
Stenting side	2 (10.0)	2 (11.8)	0.633
No. MES	1/2	1/9	
Contralateral side	1 (5.0)	0 (0.0)	0.541
No. MES	1	0	
Carotid duplex follow-up	(*n* = 13)	(*n* = 14)	
Presence of restenosis	1 (7.7%)	2 (14.3)	0.586
Present of instant thrombosis	0	0	
P2Y12 Reaction Units	(*n* = 21)	(*n* = 19)	
Improved, category (>20%)	5 (23.8)	15 (78.9)	0.001
Improved, degree	0.0 (−3.0–3.0)	21.0 (6.0–35.0)	<0.001

**Table 3:** Clinical and adverse events.

	**Clopidogrel group (*n* = 22)**	**Ticlopidine–Ginkgo group (*n* = 20)**	***P*-value**
**CLINICAL EVENTS**			
Ischemic stroke/TIA	0 (0.0)	1 (5.0)	0.090
Acute myocardial infarction	0 (0.0)	0 (0.0)	NA
All-cause mortality	1 (4.5)	0 (0.0)	0.081
**ADVERSE EVENTS**			
Hematologic adverse event^*^	0 (0.0)	0 (0.0)	NA
Procedure-related adverse event^†^	0 (0.0)	0 (0.0)	NA

* *Neutrophil < 1500, PLT < 100,000, AST/ALT >120*.

† *Puncture-site hematoma requiring surgical intervention, anemia requiring transfusion, prolongation of hospital admission*.

## Discussion

The main findings of this trial are as follows: (1) clinical events including ischemic stroke, acute myocardial infarction, and all-cause death related to carotid stenting were rare, while surrogate biomarker outcome events, detected by DWI and TCD, were highly frequent; (2) the primary outcome, new ischemic lesion on follow-up DWI, did not show significant difference between the two study groups; (3) ticlopidine–ginkgo treatment significantly improved drug resistance (P2Y12 Reaction Units). There were no safety concerns identified within this small group of patients.

During the study screening period, 45.9% (106/231) of patients treated with CAS showed clopidogrel resistance, which may be related to both genetic and non-genetic factors (e.g., concomitant use of statins, proton pump inhibitors, and presence of diabetes) (29–33). The reported prevalence of clopidogrel resistance in cerebrovascular intervention ranges from 28.8 to 65.8% (28, 34–36), and it is more prevalent in Asian individuals (37).

The failure of this study could be attributed to the following reasons. First, our study population showed a low rate of vascular events (one stroke and one death) during the follow-up period. With the advances in neurointerventional technique and the use of high-intensity statins prior to CAS, the incidence of periprocedural stroke/TIA has substantially decreased (38). Another explanation is the short duration of follow-up in this study. Most clinical trials testing the role of antiplatelet agents in patients with high on-treatment residual platelet reactivity had a long follow-up period (3, 5, 39, 40), while only peri-procedural laboratory events were measured in the present trial. The third reason is the differences in the baseline characteristics (more asymptomatic patients in the clopidogrel group) and treatment protocol (stenting with a delay of at least 4 days of randomization in the ticlopidine–ginkgo group) between the groups.

Although this trial failed to show the superiority of ticlopidine–ginkgo over standard clopidogrel therapy in patients with high on-treatment platelet reactivity undergoing CAS placement, it has some clinical implications. Our results suggest the possibility of clinical trials that use comprehensive surrogate markers of three different sites (donor, migrating, and recipient). Relatively small number of patients and a short follow-up period may be required to evaluate the efficacy of drug therapy. For example, the efficacy of antiplatelet agents has been successfully tested using MES as a surrogate marker in clinical trials with a small cohort (*n* = ~100 patients) (20, 21). In addition, a recent meta-analysis showed that 1 out of 10 patients with silent DWI lesions during invasive vascular or cardiac procedures experienced stroke or TIA (41). In the present study, several patients showed embolism on DWI or TCD measures, while only two patient showed a clinical event. In this context, surrogate biomarker outcome-based clinical studies may be helpful before starting a large clinical trial. Further clinical trials using this approach and testing other thienopyridines in various neurointerventional settings (e.g., endovascular aneurysmal repair) are warranted. Lastly, the results of this trial showed considerable improvement in P2Y12 Reaction Units without hematologic or adverse effects by using ticlopidine–ginkgo. Further studies with a larger cohort and long-term follow-up are warranted.

This study has several limitations. First, the primary outcome was the surrogate endpoint; further studies are required to evaluate the long-term clinical efficacy of ticlopidine–ginkgo. However, based on our results, it is unlikely that long-term clinical efficacy events would differ between the groups. Second, only Korean patients were included in the study; this may limit the generalizability of our results, since a high prevalence of clopidogrel resistance has been reported in the Asian population (42). Third, more symptomatic patients with carotid stenosis were enrolled in the ticlopidine–ginkgo group. Symptomatic carotid stenosis is associated with a higher risk for recurrent thromboembolism, and the imbalance in the baseline in this trial may have affected the study endpoints. Finally, 96 h waiting periods for the ticlopidine–ginkgo group could have affected the study results.

## Conclusion

Our findings indicate that a multimodal biomarker-based clinical trial is feasible in research related to clinical stroke, with more frequent endpoint events observed than those observed from clinical outcomes. Compared to continued treatment with clopidogrel, treatment with ticlopidine–ginkgo significantly improved drug resistance. No adverse effects were observed within this small group of patients. However, no difference was observed in early surrogate biomarkers between the treatment groups.

## Author Contributions

OB was the principal investigator, participated in the study design, obtained funding, and wrote the report. J-WC contributed to the data collection, data analysis, and wrote the report. SK, JH, ML, JL, K-YL, M-SP, SS, KK, and PJ contributed to data collection and interpretation and design of the study, reviewed the report, and gave scientific advice.

### Conflict of Interest Statement

The authors declare that the research was conducted in the absence of any commercial or financial relationships that could be construed as a potential conflict of interest.
