# Supplementary material for: Comparison of Clopidogrel and Ticlopidine/Ginkgo Biloba in Patients With Clopidogrel Resistance and Carotid Stenting
Source: Front Neurol. 2019 Jan 30;10:44. doi: 10.3389/fneur.2019.00044 (PMC6363652; doi:10.3389/fneur.2019.00044)
Supplement: Supplementary file 1 [file Table_1.docx]

**SUPPLEMENTAL MATERIAL**

**Comparison of clopidogrel and ticlopidine/*Ginkgo biloba* in combination with aspirin in patients with clopidogrel resistance and carotid stenting**

**CONSORT 2010 Flow Diagram**

Allocated to intervention (n=22)

♦ Received allocated intervention (n=22)

♦ Did not receive allocated intervention (give reasons) (n=0)

Excluded (n=82)

♦  Not meeting inclusion criteria, no resistance to clopidogrel (n=70)

♦  Declined to participate (n=12)

♦  Other reasons (n=0)

Allocated to intervention (n=20)

♦ Received allocated intervention (n=20)

♦ Did not receive allocated intervention (give reasons) (n=0)

## Follow-Up

Analysed (n=20)
♦ Excluded from analysis (give reasons) (n=0)

## Analysis

Analysed (n=22)
♦ Excluded from analysis (give reasons) (n=0)

Lost to follow-up (give reasons) (n=0)

Discontinued intervention (give reasons) (n=0)

Lost to follow-up (give reasons) (n=0)

Discontinued intervention (give reasons) (n=0)

## Enrollment

## Allocation

Randomized (n=42)

Assessed for eligibility (n=124)
